# Is *KCNH1* mutation related to coronary artery ectasia

**DOI:** 10.1186/s12872-019-01276-4

**Published:** 2019-12-17

**Authors:** Mohammad Rafi Noori, Bo Zhang, Lifei Pan

**Affiliations:** Department of Coronary Heart Disease, The first affiliated Hospital of Dalian Medical University, Lianhe Avenue, Dalian, China

**Keywords:** Coronary artery ectasia, Acute myocardial infarction, KCNH1, Gene mutation, Diarrhea, Embolism

## Abstract

**Background:**

Coronary artery ectasia (CAE) is an uncommon finding in patients undergoing coronary angiography and acute myocardial infarction is an extremely uncommon condition in the presence of coronary artery ectasia. To date, 50 gene variants associated with coronary artery disease have been identified, but none appear to be related to coronary artery ectasia.

**Case presentation:**

This is a rare case of Coronary artery ectasia which is considered to be related to Gene variations in *potassium voltage-gated channel subfamily H member 1, KCNH1* (encoding a protein designated ether à go-go, EAG1 or KV10.1).

**Conclusion:**

Occurrence of Acute myocardial infarction in patient with coronary artery ectasia after diarrhea is a very rare condition and involvement of KCNH1 gene mutation which is described in this case report.

## Background

Coronary artery ectasia (CAE) is defined as dilation of the coronary vascular lumen up to a diameter 1.5 times that of the adjacent normal coronary artery [1, 2]. Angina pectoris is the most common clinical manifestation [3] while acute myocardial infarction is an extremely uncommon condition in the presence of coronary artery ectasia, detected only in < 1% cases [4]. Genetic variation has been reported as one of the risk factors for coronary artery disease. To date, 50 gene variants associated with coronary artery disease have been identified [5], but none appear to be related to coronary artery ectasia.

Here, we have reported a rare case of acute myocardial infarction with coronary artery ectasia and mutation in *potassium voltage-gated channel subfamily H member 1, KCNH1* (encoding a protein designated ether à go-go, EAG1 or KV10.1) gene.

## Case presentation

A 33-years-old male was admitted into our hospital after 3 hrs of intermittent chest pain. Previously, the patient was admitted to our hospital and diagnosed with first-degree atrioventricular conduct block and coronary artery ectasia, which was conservatively treated with aspirin (100 mg/qd, atorvastatin 20 mg/qd) on a routine basis. After 7–8 episodes of diarrhea, the patient experiences a similar chest pain to previous episodes. Electrocardiography (ECG) was performed immediately, which disclosed ST elevation in inferior leads (II, III and avF). The patient was defibrillated after developing a sudden ventricular fibrillation.

**Fig. 1:**
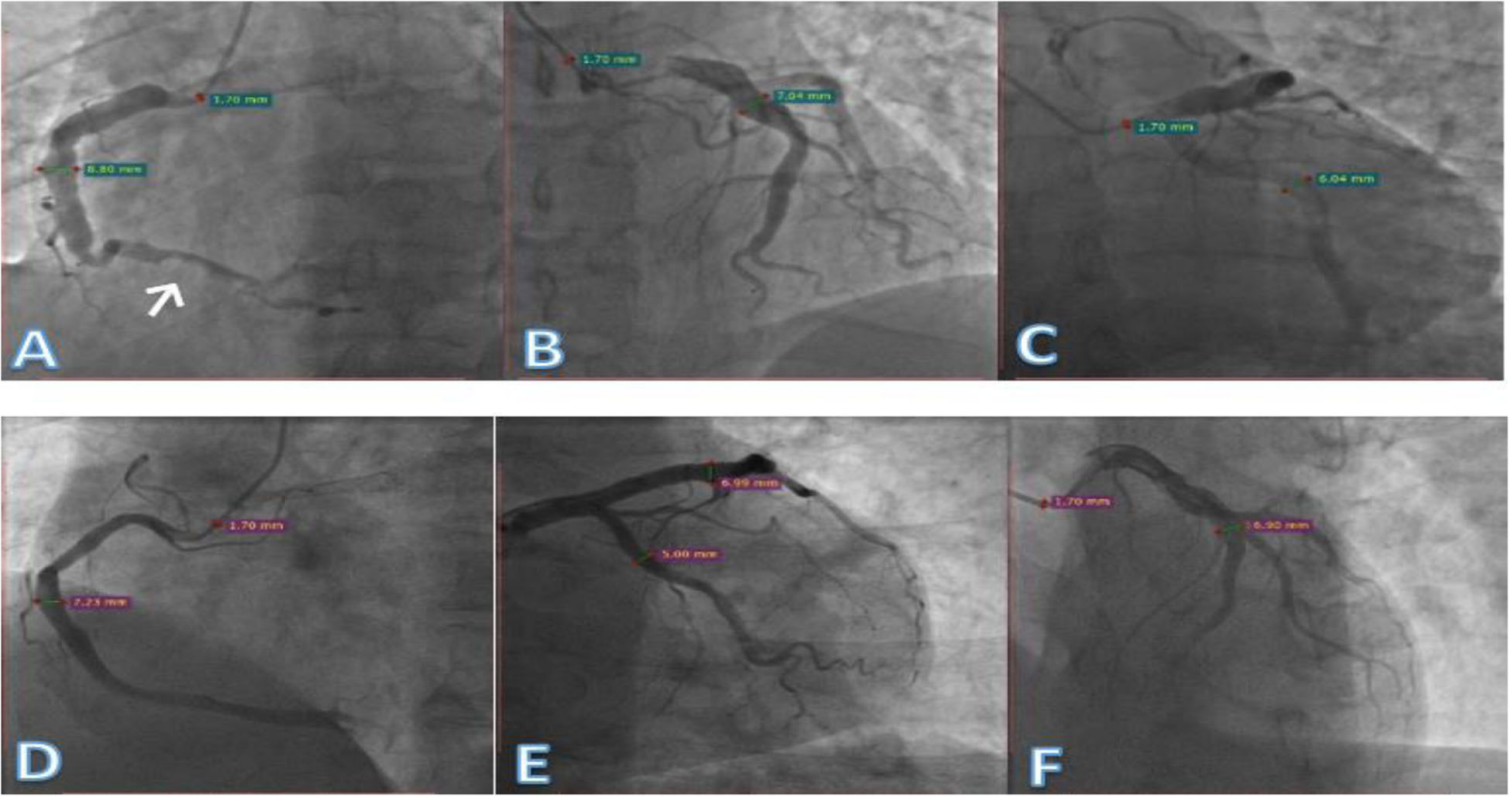
**a**: Left anterior oblique view of ectatic right coronary artery with diameter of 8.80 mm and thrombus in the distal portion of RCA (arrow). **b**, **c**: Ectasia of left anterior descending artery and left circumflex artery with diameter of 7.04 and 6.04 (respectively), in the right cranial and caudal views. **d**: Ectasia in mid to distal segment of RCA with diameter of 7.23 mm. E, F: Ectasia in proximal to mid segment of LAD with diameter of 6.99 mm

On admission, his pulse was recorded as 72/min and blood pressure (BP) as 120/70 mmHg. In laboratory examinations, cardiac enzyme contents were as follows: creatine kinase (CK)413 IU/L, high-sensitivity troponin I (hs-TnI) 3.303 μg/L, creatine kinase muscle/brain mass (CK-MBmass) 36.94 μg/L. The consequent diagnosis was acute ST elevation myocardial infarction (STEMI). The patient underwent elective coronary angiography, which revealed normal left main coronary artery (LMCA), left anterior descending (LAD) middle segment light stenosis with aneurysm-like ectasia and aneurysm-like ectasia of proximal left circumflex artery (LCX), as well as aneurysm-like ectasia of middle segment and thrombus in the distal segment of right coronary artery (RCA) (Fig. 1a-c).

Despite angiographic detection of a thrombus, conservative therapy (aspirin 100 mg/qd, ticagrelor 90 mg/tid, atorvastatin 20 mg/qd) appeared the optimal treatment choice. For further examination of cardiac function, echocardiography was performed, which revealed right and left ventricle regional wall motion abnormalities, left ventricle diameter of 55 mm, and left ventricle ejection fraction (LVEF) of 56%.

**Fig. 2:**
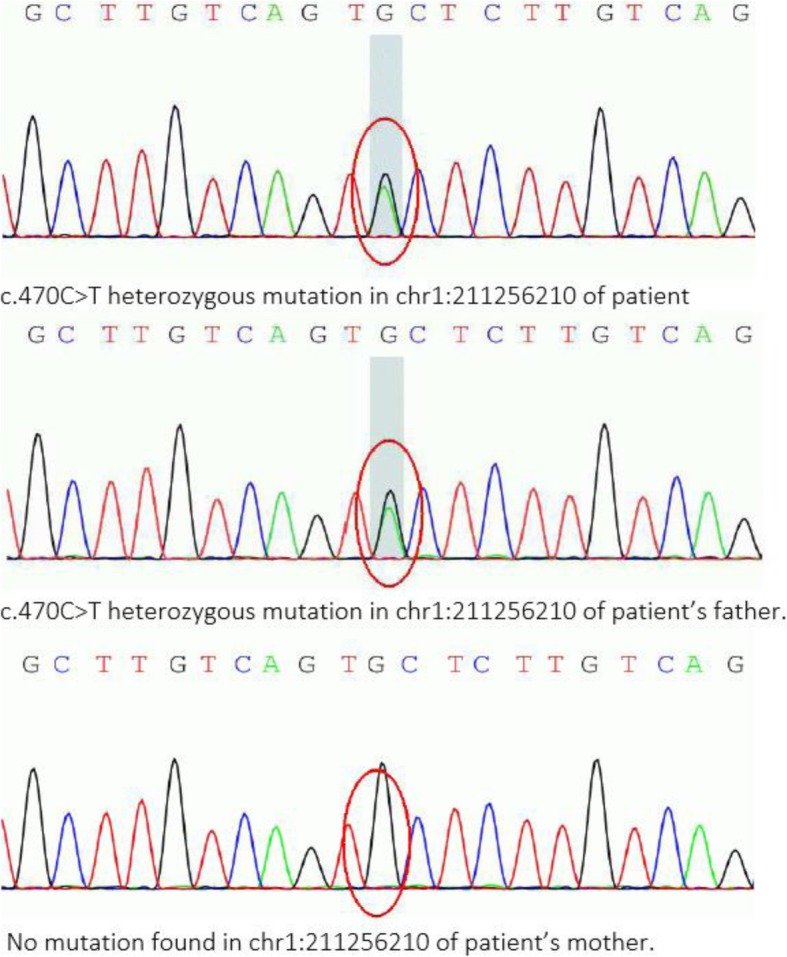
Pyrosequencing profiles of three genotypes of the c.470C > T (chr1:211256210) KCNH1 mutation were identified. The patient and his father carried the same genetic mutation but not his mother. Genetic mutational analysis was performed using www.precisionmdx.com

During history taking, the patient provided information on the medical history of his father who was admitted to another hospital due to a similar complaint of chest discomfort and underwent coronary angiography, which showed ectasia of the middle to distal segment of RCA and mid segment of LAD and normal LMCA, LCX (Fig. 1d-f). For analysis of the genetic relationships, high throughput sequencing testing was conducted, as depicted in (Fig. 2).

**Fig. 3:**
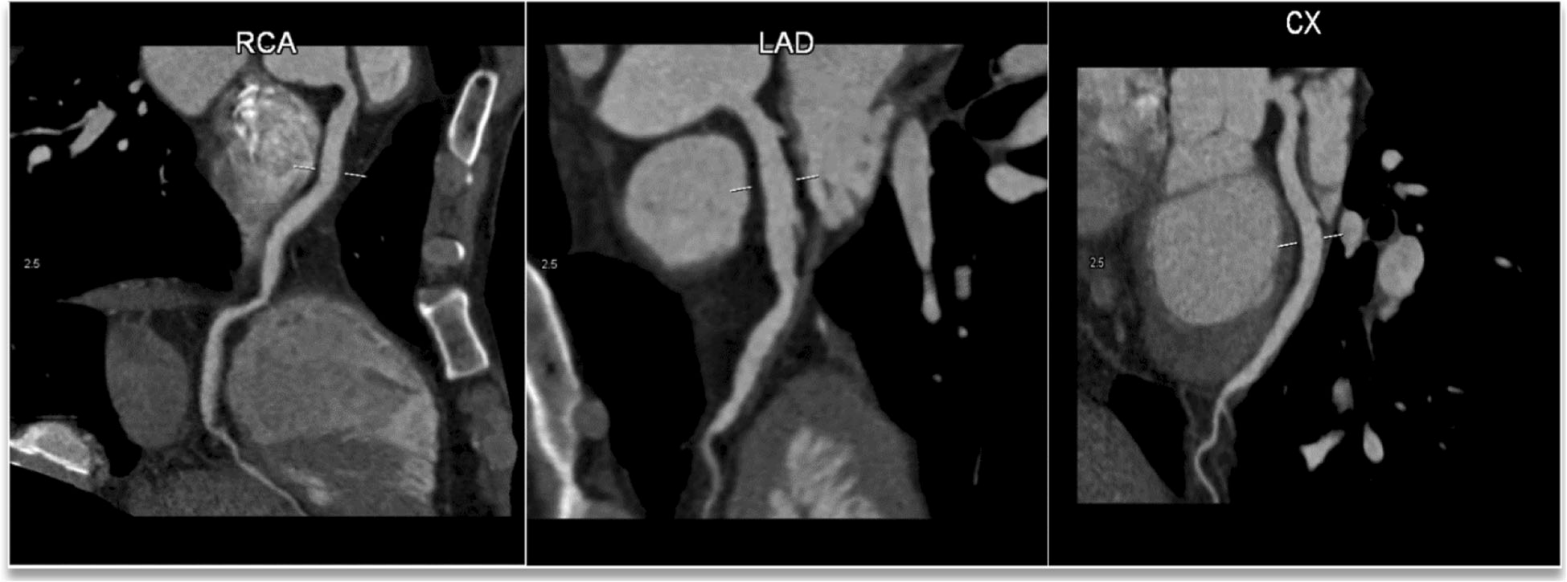
Right coronary artery with a diameter of 8.80 mm, left anterior descending artery with a diameter of 7.04 mm, left circumflex artery with a diameter of 6.04 mm

After 1 year of conservative therapy, the patient was re-admitted to our hospital due to short episodes of chest pain, which usually ended within a few seconds. Computed tomography angiography (CTA) revealed normal LMT, slight calcification and ectasia of LAD, LCX, and RCA (Fig. 3). Rivaroxaban 10 mg Qd was selected for anticoagulant therapy, along with atorvastatin 20 mg/qd.

## Discussion

CAE is only observed in 5% patients undergoing coronary angiography. Overall, ~ 20–30% CAE cases are congenital, with the remainder being acquired, and up to 20% acquired CAE is attributed to atherosclerosis, which is mostly associated with obstructive coronary artery disease [6]. Congenital CAE is mainly linked to cardiac anomalies, such as bicuspid aortic valve, aortic root dilation, ventricular septal defect or pulmonary stenosis [6, 7]. Acquired CAE association with inflammatory or connective tissue disorder constitutes only 10–20% cases, such as scleroderma Elhers-Danlos syndrome, anti-neutrophil cytoplasmic antibody-related vasculitis syphilitic aortitis and Kawasaki disease [8]. All the above etiologies of coronary artery ectasia were excluded based on patient history and serological test findings.

Disturbances in blood flow filling and washout are an inherent characteristic of CAE [9]. which may be the basis of ischemia or ischemic events in this patient group, and can alter the incidence of exercise-induced angina pectoris or myocardial infarction regardless of the severity of coexisting stenotic lesions. Slow blood flow to ectatic segments of coronary artery is reported to induce activation of platelets, coagulation and thrombus formation [10].

Before admission to the hospital, the patient experienced several episodes of diarrhea, which could cause dehydration (loss of body fluid), leading to higher concentrations of chemicals circulating in blood and increased serum osmolality, culminating in ectatic artery and consequent disturbance in blood flow, in turn, triggering formation of thrombus in the right coronary artery. After a year of follow-up, coronary CT angiography confirmed absence of thrombus in RCA. Accordingly, anticoagulant therapy with rivaroxaban 10 mg alone was selected for treatment.

*KCNH1* (encoding ether à go-go, EAG1 or KV10.1), a voltage-gated potassium channel, is predominantly expressed in the central nervous system (CNS) [11].and its overexpression provides growth advantages to cancer cells and enhances proliferation. Mutations in the *KCNH1* gene are reported to be associated with Zimmerman-Laband syndrome and Temple-Baraitser syndrome, characterized by intellectual disability, epilepsy, hypoplasia abnormal muscle tone and craniofacial dysmorphology [12]. Epilepsy is the main manifestation of *KCNH1* mutation-induced disorders. However, our patient had none of the above features or epileptic history.

To our knowledge, this is the first documented report linking *KCNH1* mutation to coronary heart disease. The detection of mutations in both family members suggests that *KCNH1* abnormalities are significantly involved in this condition. Further studies focusing on the *KCNH1* gene and the mechanisms underlying its specific association with coronary artery disease are thus warranted.

## Data Availability

All data generated during this study are included in this published article and its supplementary information files**.**

## Abbreviation

BP: Blood pressure
CAE: Coronary artery ectasia
CK: Creatine kinase
CK-MBmass: Creatine kinase muscle/brain mass
CNS: Central nervous system
CTA: Computed tomography angiography
ECG: Electrocardiography
hs-TnI: High-sensitivity troponin I
*KCNH1*: Potassium voltage-gated channel subfamily H member 1
LAD: Left anterior descending
LCX: Left circumflex artery
LMCA: Left Main coronary artery
LVEF: Left ventricle ejection fraction
RCA: Right coronary artery
STEMI: ST elevation myocardial infarction
